# Self-Care and Pathophysiological Function in Patients with Chronic Heart Failure

**DOI:** 10.1007/s12529-019-09822-2

**Published:** 2019-11-21

**Authors:** Dionne Kessing, Johan Denollet, Jos Widdershoven, Nina Kupper

**Affiliations:** 1 Center of Research on Psychological and Somatic disorders (CoRPS), Department of Medical & Clinical Psychology, Tilburg University, PO Box 90153, 5000 LE Tilburg, The Netherlands; 2 Department of Cardiology, Elisabeth-TweeSteden hospital, Tilburg, The Netherlands

**Keywords:** Adherence, Self-care, Anemia, Renal function, Inflammation, Heart failure

## Abstract

**Background:**

Self-care is assumed to benefit physiological function associated with prognosis in patients with chronic HF, but studies examining these relations are lacking. This study aims to prospectively examine the association of self-reported HF self-care with HF-associated pathophysiological markers, including renal, hematological, and immune function.

**Method:**

Patients with chronic HF (*n* = 460, 66.2 ± 9.6 years, 75% men) completed questionnaires and provided blood samples at baseline and 12-month follow-up. Linear mixed models examined random intercept and fixed between- and within-subjects effects of global self-care and the individual self-care behaviors on log-transformed TNF-α, IL-6, and IL-10, the glomerular filtration rate of creatinine (GFR_creat_), and hemoglobin (Hb), controlling for sociodemographic and clinical covariates.

**Results:**

Self-care was independently associated with lower GFR_creat_ levels (*β* = − .14, *P* = .023) and improvement in self-care with a reduction in GFR_creat_ (*β* = − .03, *P* = .042). Individual self-care behaviors were differentially associated with renal, inflammatory, and hematological markers. Regular exercise was associated with level differences in IL-6 (*P* < .001), and improvement in exercise was associated with increasing GFR_creat_ (*P* = .002) and increasing Hb (*P* = .010). Fluid restriction was associated with lower overall GFR_creat_ (*P* = .006), and improvement in fluid restriction was associated with decreasing GFR_creat_ (*P* = .014). Low-sodium intake was associated with lower levels of Hb (*P* = .027), lower TNF-alpha (*P* = .011), and lower IL-10 (*P* = .029). Higher levels of medication adherence were associated with reduced pro-inflammatory activation (*P* < .007).

**Conclusion:**

Our findings suggest that better global self-care was associated with poorer renal function. Performing self-care behaviors such as regular exercise and medication adherence was associated with improved physiological functioning, while restriction of fluid and sodium, and the associated daily weight monitoring were associated with adverse levels of pathophysiological biomarkers.

## Introduction

Chronic heart failure (HF) is among the leading causes of mortality in developed countries, affecting about 15 million people across Europe [1, 2]. HF prevalence is expected to increase due to the aging of the population, placing a significant clinical and economic societal burden [3]. Despite therapeutic advances showing the ability to reduce admission rates for HF [4, 5], effective management of HF remains challenging for both health care providers and patients. In addition to medical treatment, patients with HF are recommended to perform a complex regimen of multiple self-care behaviors (i.e., sodium and fluid restriction, regular exercise, medication adherence, monitoring and consulting for HF symptoms) [6].

It is assumed that HF self-care is associated with better health outcomes, but the available evidence is inconsistent [7]. Potential mechanisms have been proposed suggesting that self-care benefits HF outcomes via neurohormonal and inflammatory function [8]. To date, only one cross-sectional study on 168 patients with HF has been published showing an association between self-care management and reduced myocardial stress and systemic inflammation [9]. Intriguingly, those reporting to be highest in self-care management were at increased risk for myocardial stress and inflammation. However, findings were cross-sectional and restricted to self-care management; no associations were found for other elements of self-care such as dietary restrictions.

Emerging evidence shows the prognostic importance of cardiorenal and hematological function in patients with HF [10]. Due to chronic volume overload, HF is associated with progressive renal dysfunction, impaired renal perfusion, and excessive production of vasoconstrictive neurohormones [11]. Renal dysfunction and associated elevated inflammation may lead to a decrease in erythropoietin levels and reduced hematopoietic proliferation, evolving into anemia [12]. Chronic anemia is associated with reduced tissue oxygenation, which, in HF patients, results in hemodynamic compensatory responses to enhance oxygen-carrying capacity, thereby worsening cardiac function. As self-care behaviors such as fluid and salt restriction intend to reduce volume overload and avoid decompensation, self-care might affect three important mechanistic pillars of HF disease progression, i.e., inflammation, renal dysfunction, and anemia.

No prior study has examined the longitudinal relationship between self-care and these pathophysiological markers of HF disease severity [13]. Therefore, we aimed to prospectively examine the association of self-reported HF self-care with systemic inflammation (i.e., serum levels of tumor necrosis factor alpha (TNF-α), interleukin-6 (IL-6), and IL-10), renal function (i.e., estimated glomerular filtration rate of creatinine (GFR_creat_), and hemoglobin (Hb), respectively) at inclusion and 1-year follow-up. We hypothesized that poorer self-care would be associated with increased inflammation, poorer renal function, and more anemia, as these behaviors are directed at promoting allostatic balance [6]. Moreover, based on the argumentation set out above, we expected the pathophysiological markers to be differentially associated with individual self-care behaviors, as exercise affects different pathophysiological pathways than fluid and salt restriction. We expected exercise to be associated with altered inflammatory markers [14], while limiting fluids and salt intake should be associated with improved renal and hemoglobin function [15].

## Methods

### Study Population and Design

Consecutive outpatients with chronic HF were recruited from two cohorts from three general hospitals in the Netherlands. The design and procedure of patient inclusion have been published previously [16, 17]. Inclusion criteria were as follows: a confirmed diagnosis of HF with a reduced left ventricular ejection fraction (LVEF) ≤ 40%, New York Heart Association (NYHA) function classes I–III, stable on HF medication, and age ≤ 80 years. Exclusion criteria were as follows: a hospital admission 1 month prior to inclusion, other life-threatening conditions with a life expectancy < 1 year, severe psychiatric comorbidity (except for anxiety and/or depression), severe cognitive impairment, or insufficient competence of the Dutch language [16, 17]. The cardiologists or HF nurses approached eligible patients for participation during an outpatient clinic visit. An independent study investigator called invited patients to schedule a baseline appointment in which they were given additional information about the study protocol before signing informed consent. At baseline and at 12-month follow-up, participants completed questionnaires at home to assess socio-demographic and psychological variables including HF self-care that were returned within 2 weeks by mail in a stamped and pre-addressed envelope. Questionnaires were checked for completeness. Participants were contacted in the event of a missing questionnaire or items. Venous blood samples were drawn for cytokine measurement and clinical laboratory values during daytime in a planned outpatient clinic visit at baseline and 12-month follow-up. Participants were instructed not to exercise, smoke, or drink prior to their blood draws and were told to reschedule when ill (cold or fever).

In total, 709 patients were eligible to participate. Data originated from two observational cohort studies for which ethics approval was obtained from the relevant medical ethics committees. The investigation conforms to the principles outlined in the Declaration of Helsinki (2013).

### Self-Care

The 9-item European Heart Failure Self-care Behavior scale (EHFScB-9) [16] assessed self-care at baseline and 12-month follow-up. Items were rated on a 5-point Likert scale from 1 (“I completely agree) to 5 (“I completely disagree”). Item scores were reversed in order to calculate sum scores (range 9–45). Scores were then transformed into a standardized score from 0 to 100 [18, 19], with higher scores reflecting better self-care. The EHFScB-9 includes a 4-item “consulting behavior” subscale measuring whether patients contact their doctor/nurse in case of shortness of breath, ankle swelling, weight gain, and fatigue. The internal consistency of the total self-care scale and the consultation behavior scale in the current database at baseline and 12-month follow-up was good, with Cronbach’s *α* = .79 for the total scale, *α* = .86 for the consulting scale. Test retest reliability for these two scales was moderate with the intra-class correlation being .66 for the total score and .60 for the consultation behavior scale. In addition to the two scale scores, scores on individual self-care behaviors (single items: weight monitoring, fluid restriction, salt restriction, medication adherence and regular exercise) were analyzed. Intra-class correlations were .61 for daily weight monitoring, .47 for fluid restriction, .55 for salt restriction, .65 for regular exercise, and .11 for medication adherence (potential ceiling effect: 310 of the 391 patients with follow-up data scored the maximum score of 5 on the medication adherence item at both time points). These ICC results suggest that there is a moderate amount of stable between- and within-person variance, leaving sufficient room for a reliable change prediction.

To be able to discriminate between between- and within-subjects effects, for all self-care scores, we calculated a person mean, reflecting the average level of self-care, consultation behavior, or adherence to specific health behaviors across time. This variable was used to assess between-subject differences. We also calculated the time-specific deviation from the person mean to use as the independent variable for within-subjects effects.

### Socio-demographic and Clinical Characteristics

Socio-demographic variables were assessed at baseline using purpose-designed items including educational level (primary school or less vs. > 8 years of education), current smoking, marital (alone vs. having a partner), and employment status (yes/no). Patients’ medical records were searched for information on body mass index (BMI), disease characteristics (i.e., (ischemic) etiology, LVEF, NYHA function classes I–II vs. III), cardiac history of previous myocardial infarction, percutaneous coronary intervention, or coronary artery bypass graft surgery, and pharmaceutical treatment (e.g., beta-blockers, angiotensin-converting enzyme inhibitors, angiotensin receptor blockers, diuretics, and aspirin). The presence of the following medical comorbidities was documented: diabetes, chronic obstructive pulmonary disease, peripheral arterial disease, hypertension, hypercholesterolemia, gastrointestinal disease, cancer, cerebrovascular disease including transient ischemic attack, liver disease, and renal failure.

### Measurements

Venous blood samples were drawn at baseline and at 12-month follow-up. Procedures have extensively been published before [20, 21]. Renal function was measured according to the K/DOQI guidelines [22, 23] by calculating the glomerular filtration rate of creatinine. The MDRD equation defined as GFR_creat_ < 60 ml/min per 1.73 m^2^ was used to define renal dysfunction [20]. Inflammatory biomarker data (TNF-α, IL-6, and IL-10) were available for a subsample, as the blood substudy started later, and obtained using standard hospital protocol. Patients with rheumatic arthritis or gout were not eligible for the cytokine analyses, due to the chronic inflammation characteristic of these comorbidities. Blood was allowed to clot at room temperature for at least 20 min and centrifuged. Aliquoted serum samples were stored at − 80 °C in anticipation of further processing. Concentrations of IL-6 (sensitivity: 2 pg/ml), IL-10 (sensitivity: 1 pg/ml), and TNF-α (sensitivity: 1.7 pg/ml) were measured using a solid-phase, enzyme-labeled, chemiluminescent immunometric assay (Immulite 1000, Siemens Healthcare Diagnostics Breda, the Netherlands). All tests were measured in accordance with the manufacturer’s recommendations. The intra-assay variation was less than 10%, and the inter-assay variation less than 11%. Plasma hemoglobin (Hb) was determined using the Siemens ADVIA 120 Hematology system in the hospitals’ central Clinical Chemistry & Hematology Laboratory as an indicator of anemia [21]. Continuous levels of Hb were used in the analyses.

### Statistical Analysis

All biomarkers were tested for outliers of > 3 SDs, which were considered erroneous and thus removed (Hb: T0: *n* = 1, T12m: *n* = 1; GFR_creat_: T0: *n* = 2, T12m: *n* = 1; TNF-alpha: T0: *n* = 3, T12m: *n* = 4; IL-6: T0: *n* = 4, T12m: *n* = 4, IL-10: T0: *n* = 11, T12m: *n* = 11 (both including below detection limit)). Prior to running analyses, skewed data were log-transformed. Group differences in baseline characteristics were analyzed for (a) patients with and without complete data at baseline/12-month follow-up and for (b) patients with and without biomarker data on both time points using Student’s *t* tests for independent samples and chi-square tests (Fischer’s exact test when appropriate) for continuous and discrete variables, respectively. Descriptive baseline statistics were calculated and correlated with baseline self-care by calculating Pearson (continuous variables or Spearman (dichotomous variables)) correlations. Repeated measures ANOVA analyses were run to establish whether self-care behavior significantly changed from baseline to 12 months follow-up. Pearson correlations were calculated for baseline levels of GFR_creat_, Hb, TNF-α, IL-10, and IL-6 with individual self-care behaviors at baseline and at 12 months follow-up.

For the main analyses, separate linear mixed models with maximum likelihood estimation (Satterthwaite approximation for df calculation (standard in SPSS)) were used to examine longitudinal associations of the person mean of continuous self-care (i.e., average of the two measurement occasions; to gauge between-subject effects) and the time-specific deviation of the self-care score from the person mean (reflecting within-subject differences) with continuous levels of pathophysiological markers (dependent variables with repeated measures) of renal and hematological function and inflammation at baseline and 12-month follow-up. The linear mixed modeling technique is suitable for analysis of repeated measurements, as it takes the possibility of correlated data into account. In addition, in contrast to traditional repeated measures ANOVA, one missing measurement occasion does not automatically lead to exclusion of that patient from analysis, limiting bias and preserving statistical power. Another advantage to linear mixed modeling analysis is the possibility of measuring variables as fixed variables or as time-varying variables. Self-care was added as a time-varying predictor. We added a random intercept to gauge to potential effect of important unmeasured explanatory variables. As an indication of effect size, standardized estimates (*β*) were calculated for main self-care effects and their interactions in excel using the standard deviations (DV, IV) and raw estimates. In the first models, time and self-care (between and within) were added as predictors of the level of each pathophysiological marker across time. Second, we adjusted for a priori chosen socio-demographic and health behavioral fixed covariates, chosen for their established association with the pathophysiological marker of interest and/or self-care, including age, sex, partner status, education, current smoking status, BMI, and diabetes [12]. Then, we adjusted for HF disease severity (NYHA function class and LVEF), time since diagnosis in years, and prescribed medication (i.e., angiotensin-converting enzyme inhibitors or angiotensin receptor blockers, beta-blocking agents, and diuretics for GFR_creat_ and Hb [12]; aspirin and beta-blocking agents for inflammatory cytokines). Finally, we added the interaction of self-care with time to gauge within-subject effects of self-care on the course of the biomarkers. To examine the relative contribution of each self-care behavior to the concentrations of pathophysiological markers, multivariable linear mixed models were repeated with the person mean and deviation score over time of the consultation subscale and the individual self-care item scores for each pathophysiological outcome as a time-varying dependent variable. Resulting estimates indicate the change in the dependent variable when the independent variable increases with one point, and equals *B*. A *P* value < .05 was considered of statistical significance. To reduce the chance of false discovery by multiple testing (subscale and individual behaviors), we applied the Benjamini-Hochberg procedure to the *P* values [24]. All analyses were performed using IBM SPSS Statistics version 24 (IBM Corp. Released 2016. IBM SPSS Statistics for Windows, Version 24.0. Armonk, NY: IBM Corp.).

## Results

### Sample Characteristics

**Fig. 1:**
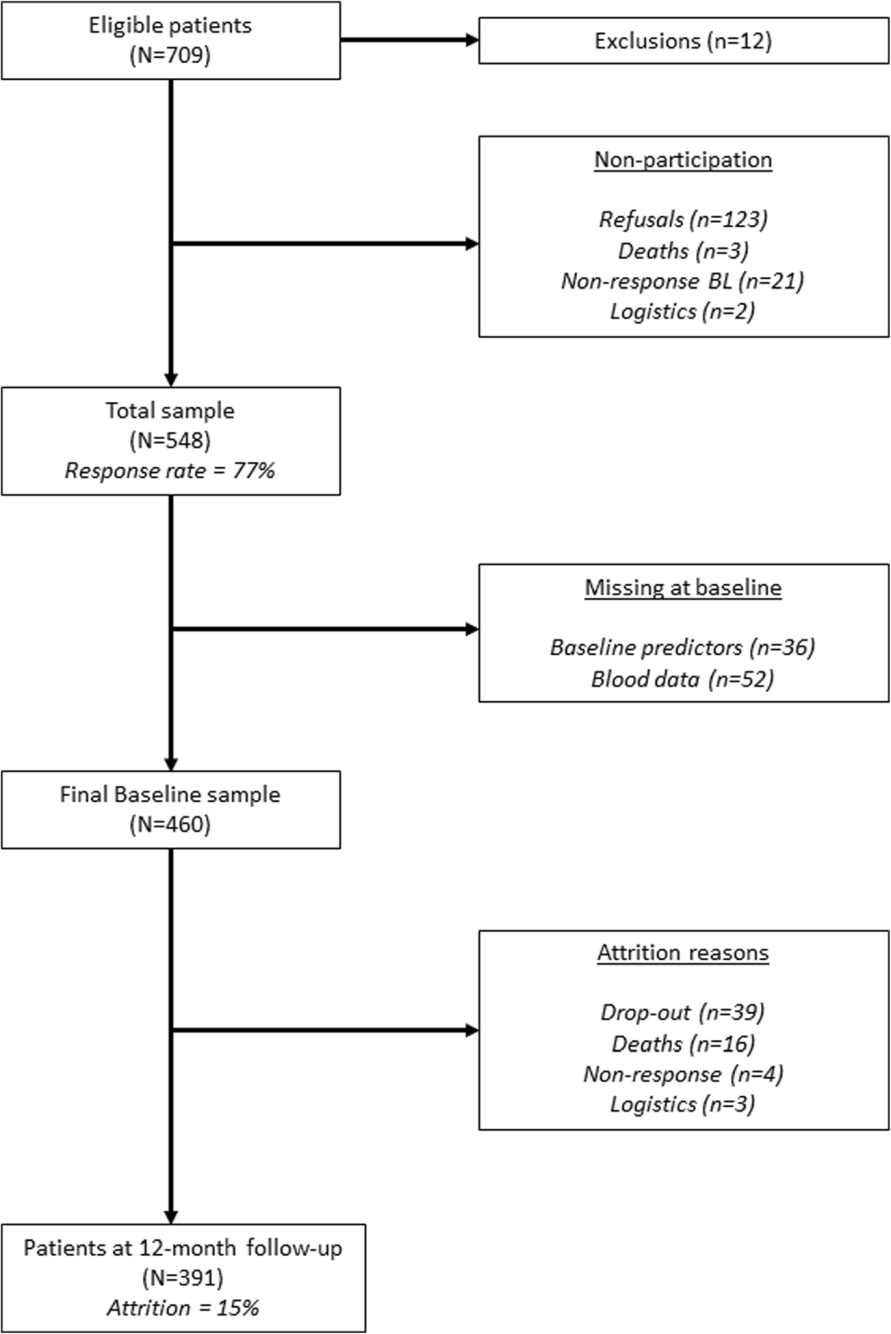
Flowchart of inclusion

A flowchart of inclusion is presented in Fig. 1. In total, 709 patients were eligible and invited to participate in the study of which 548 patients (77%) finally participated. Blood collection at baseline was not available for 52 participants due to delay in the start of the blood collection sub study, and 36 participants were excluded due to missing data on any of the baseline clinical variables or questionnaires. The final baseline survey sample for the current paper thus included 460 participants (mean age 66.2 ± 9.6 years, 75% male sex) with complete data at baseline. There were no differences in responders vs. non-responders in socio-demographic baseline characteristics, nor were there differences between included and excluded (*n* = 88) patients in any of the socio-demographic characteristics. With respect to clinical characteristics, excluded patients were characterized by a poorer LVEF (30% vs. 32%, *P* = .016) and increased prescription of diuretics (85% vs. 73%, *P* = .016) compared with included patients.

At follow-up, we retained 391 patients with survey and clinical data, indicating an overall attrition rate of 15%. GFR_creat_ was available for 448 patients at baseline, and 391 patients at follow-up (13% loss to follow-up). Data on inflammatory biomarkers were available in a subsample of 316 patients at baseline and 226 patients at follow-up (28% loss to follow-up/outliers/below detection limit). Patients with valid inflammatory markers present were characterized by a higher LVEF (32% vs. 30%, *P* < .001) and a better NYHA function class (I–II class: 81% vs. 50%, *P* < .001), but more likely to have COPD (20% vs. 13%, *P* = .025) compared with patients for whom no inflammation data was available.

Patients with complete baseline and follow-up questionnaire data were in better condition, indicated by less NYHA class III participants (45% vs. 28%; *χ*^2^ = 19.48, *P* < 001) and a lower comorbidity burden (diabetes: 35% vs. 26%, *χ*^2^ = 3.38, *P* = .07; COPD: 24% vs. 15%, *χ*^2^ = 3.82, *P* = .051; kidney disease: 45% vs. 32%; %; *χ*^2^ = 3.89, *P* = .048), compared to patients who dropped out. Patients with complete baseline and follow-up questionnaire data did not differ from patients who dropped out with respect to baseline global self-care scores, consultation behavior scores, and individual behaviors at baseline.

Socio-demographic and clinical baseline characteristics of the final sample are presented in Table 1, including correlations with baseline self-care. Better baseline self-care was associated with the presence of ischemic etiology, a shorter time since diagnosis, absence of cardiac history, the presence of diabetes, and the use of diuretics. Moreover, better self-care was associated with participation in cardiac rehabilitation, non-smokers, and lower BMI. Table 2 shows the averages of the two standardized self-care scores (global and consultation) and the five individual self-care behaviors. The averages for global self-care and consultation behavior were a bit higher at 12 months follow-up, and not significantly so, but the standard deviations were large, suggestive of large variation. Scores for individual behaviors were similar for the two time points. Splitting the group into patients who improved their self-care behaviors, patients whom had equal scores at both time points, and patients with declined in their reported self-care showed important insights. Patients with a decline in reported global self-care showed significant (all *P* < .01) reductions in all aspects of self-care. With respect to the individual self-care behaviors, the reduction seems most pronounced in daily weight monitoring and salt restriction. Patients who increased their self-care behavior (i.e., improved scores) also improved significantly across the board (all *P* < .01). With respect to individual behaviors, daily weight monitoring and fluid and salt restriction contributed most to the improvement. Table 3 shows change in the non-transformed levels (descriptive purposes) of pathophysiological outcome markers for patients who improved, deteriorated, and remained at an equal level of self-care.

**Table 1:** Baseline characteristics for total sample and correlations with global self-care

	Total (*n* = 460)	Correlation with baseline self-care	*P* value
Sociodemographic variables			
Age, mean ± SD (years)	66.2 ± 9.6	.01	.869
Male gender	344 (75)	.07	.120
Having a partner	345 (75)	*.07*	*.099*
Currently employed	67 (15)	− .06	.136
Low education*	157 (34)	*.07*	*.085*
Clinical variables			
Etiology (ischemic)	271 (59)	**.09**	**.042**
Time since diagnosis (years)^‡^	4.5 ± 4.8	**− .12**	**.011**
LVEF, mean ± SD (%)	31.8 ± 6.8	− .01	.773
NYHA class III	146 (32)	− .02	.683
Cardiac history^†^	288 (63)	**− .11**	**.009**
Hyperlipidemia	268 (58)	.02	.731
Hypertension	195 (42)	*.07*	*.096*
Peripheral arterial disease	67 (15)	.02	.570
Diabetes mellitus	122 (27)	**.10**	**.017**
COPD	76 (17)	− .01	.854
Kidney disease	55 (12)	*.08*	*.051*
Stroke/transient ischemic attack	68 (15)	.03	.562
Implantable device (yes)	70 (16)	.03	.473
Health behaviors			
Currently smoking	108 (24)	**− .13**	**.003**
BMI, mean ± SD	28.0 ± 5.1	**− .11**	**.015**
Cardiac rehabilitation	36 (8)	**.11**	**.009**
Prescribed medications			
Beta-blockers	303 (66)	*.08*	*.070*
ACEi	314 (68)	.02	.570
ARBs	115 (25)	.05	.301
Calcium antagonists	58 (13)	.05	.259
Oral anticoagulants	237 (52)	.03	.560
Statins	258 (56)	.02	.614
Diuretics	336 (73)	**.13**	**.003**
Nitrates	148 (32)	.01	.880
Aspirin	183 (40)	− .05	.275
Psychotropic medication	72 (16)	− .03	.639

Data are presented as *n* (%) unless stated otherwise. The second and third columns hold the Pearson (continuous variables) and Spearman (dichotomous variables) correlations, with baseline self-care, and accompanying *P* values. Boldfaced numbers represent significant differences (*P*<.05), while italic numbers represent trends (*P*<.10).

*ACEi* angiotensin-converting enzyme inhibitors, *ARBs* angiotensin receptor blockers, *BMI* body mass index, *COPD* chronic obstructive pulmonary disease, *LVEF* left ventricular ejection fraction, *NYHA* New York Heart Association, *SD* standard deviation, *N* may vary slightly per variable due to missing data

*Primary school or less

^†^History of myocardial infarction, percutaneous coronary intervention, or coronary artery bypass graft surgery

^‡^*N* = 36 missing values

**Table 2:** Descriptive information on self-care behavior

	Baseline (*N* = 460)	12 months FU (*N* = 391)	Improved self-care (*N* = 184)		Equal self-care (*N* = 41)		Reduced self-care (*N* = 186)	
			BL	12mo	BL	12mo	BL	12mo
Standardized scores (0–100, with higher scores signifying better adherence)								
Total self-care score	62.3 (21.6)	64.4 (19.9)	54.7 (20.3)	70.0 (17.0)	67.5 (23.6)	67.5 (23.6)	72.2 (18.0)	58.9 (20.0)
Consultation behavior score	60.5 (31.3)	64.0 (30.0)	51.6 (30.5)	71.4 (26.1)	66.6 (33.5)	65.5 (34.5)	74.2 (25.1)	56.8 (30.6)
Individual behaviors (lower scores = better adherence)								
Weight monitoring	3.6 (1.5)	3.5 (1.5)	3.8 (1.4)	3.3 (1.5)	3.3 (1.6)	3.2 (1.7)	3.2 (1.6)	3.8 (1.5)
Fluid restriction	2.4 (1.4)	2.3 (1.4)	2.8 (1.4)	2.2 (1.3)	2.1 (1.3)	2.0 (1.3)	1.9 (1.1)	2.3 (1.4)
Salt restriction	2.4 (1.4)	2.4 (1.4)	2.8 (1.4)	2.2 (1.3)	2.2 (1.4)	2.2 (1.5)	2.1 (1.3)	2.7 (1.4)
Medication adherence	1.3 (.8)	1.2 (.6)	1.4 (.9)	1.1 (.2)	1.2 (.6)	1.2 (.6)	1.1 (.4)	1.4 (.8)
Regular exercise	2.6 (1.3)	2.6 (1.3)	2.8 (1.3)	2.6 (1.3)	2.6 (1.4)	2.6 (1.5)	2.5 (1.4)	2.7 (1.3)

Improved self-care indicates higher global self-care levels at 12 months follow-up, equal self-care indicates the group patients with the same global self-care score at both time points, and reduced self-care indicates lower global self-care levels at 12 months follow-up

**Table 3:** Descriptive information on biomarkers at baseline and change at follow-up

	Patients who improved in overall self-care (*N* = 158)	Patients with stable overall self-care (*N* = 36)	Patients who reduced in overall self-care (*N* = 151)
GFR_creat_ level (ml/min/1.73m^2^)	67.1 (21.7)	69.5 (20.2)	71.4 (23.3)
ΔGFR_creat_ (ml/min/1.73m^2^)	− 1.5 (14.8)	.2 (9.9)	.02 (14.1)
Hb level (g/dl)	8.7 (.9)	8.8 (.7)	8.7 (.9)
ΔHb (g/dl)	− .03 (.7)	.1 (.8)	− .1 (.9)
TNF-ɑ level (pg/ml)	14.4 (9.9)	14.4 (8.6)	12.8 (6.8)
ΔTNF-ɑ (pg/ml)	1.8 (19.6)	5.7 (15.5)	.36 (4.7)
IL-6 level (pg/ml)	5.3 (4.4)	4.5 (2.7)	4.8 (4.1)
ΔIL-6 (pg/ml)	− .11 (4.3)	− 1.6 (4.0)	.34 (5.4)
IL-10 level (pg/ml)	2.8 (2.4)	2.2 (1.2)	2.5 (2.5)
ΔIL-10 (pg/ml)	.36 (2.1)	− .16 (1.9)	.51 (2.4)

### Global Self-Care and Pathophysiological Outcomes

Linear mixed modeling results showed that GFR_creat_ levels did not change significantly over time (*F*(1,311.05) = .42, *P* = .518) (Table 4). In unadjusted analysis, higher (i.e., better) levels of total self-care were associated with a lower GFR_creat_ (*F*(1,369.87) = 6.43; *β* = − .15, *P* = .012), indicative of an overall poorer renal function. Within-subject increase in overall self-care over the 1-year period was related to a slightly poorer renal function as well (*F* = (1310.65) = 4.06; *β* = − .03, *P* = .045). Adding baseline socio-demographic and health behavioral covariates did not affect the results. In the fully adjusted model, better overall self-care and increase in self-care over the follow-up period both remained associated with lower GFR_creat_ (*F*_between_(1364.50) = 5.19; *β* = − .14, *P* = .023) and decreasing GFR_creat_ (*F*_within_(1306.42) = 4.19; *β* = − .03, *P* = .042) respectively, beyond clinical covariates. Significant covariates of decreased GFR_creat_ were higher age, lower LVEF, and prescribed diuretics. Finally, adding the interactions of time with the person mean of self-care and its time-specific deviations resulted in the observation that in addition to their main effects, there was a trend interaction of the person mean of self-care with time (*F*(1,306.97) = 3.45, *β* = .03, *P* = .06, *B* = − .08). In this final adjusted model, the random intercept was significant (Wald = 12.06, *P* < .001) and of substantial size (variance estimate = 928.96, se = 73.61), suggesting there remain important other variables that determine the level of GFR_creat_ that were not included in our study.

**Table 4:** Longitudinal associations of total self-care and continuous levels of GFR_creat_ and hemoglobin

	GFR_creat_			Hemoglobin		
	*B*	*t*	*P*	*B*	*t*	*P*
Time	− .42	− .48	.632	− .06	− 1.30	.195
**Model 1**						
Self-care between	**− .24**	**− 2.54**	**.012**	− .003	− 1.29	.198
Self-care within	**− .09**	**− 2.01**	**.045**	*.004*	*1.76*	*.079*
Time	− .51	− .65	.518	− .06	− 1.45	.149
**Model 2**						
Self-care between	**− .21**	**− 2.33**	**.020**	− .002	− .83	.408
Self-care within	**− .09**	**− 2.04**	**.042**	*.005*	*1.81*	*.071*
Time	− .52	− .66	.512	− .07	− 1.57	.117
Age	**− 1.19**	**− 6.43**	**< .001**	*− .009*	*− 1.83*	*.069*
Male gender	4.11	1.03	.303	**.60**	**5.86**	**< .001**
Partner status (yes)	− 2.08	− .53	.600	.05	.49	.624
Education	.27	.10	.922	.07	.76	.447
Smoking	− .78	− .19	.846	*.18*	*1.72*	*.086*
BMI	− .45	− 1.30	.194	.02	1.88	.061
**Model 3**						
Self-care between	**− .21**	**− 2.28**	**.023**	− .001	− .44	.662
Self-care within	**− .09**	**− 2.04**	**.042**	*.005*	*1.79*	*.075*
Time	− .48	− .60	.547	.06	1.32	.190
Age	**− 1.11**	**− 6.08**	**< .001**	− .006	− 1.28	.202
Male gender	4.17	1.06	.289	**.55**	**5.51**	**< .001**
Partner status (yes)	2.88	.84	.460	.02	.16	.876
Education*	− .01	− .002	.998	.11	1.26	.208
Smoking	.77	.19	.846	.15	1.48	.140
BMI	− .42	− 1.22	.223	**.02**	**2.77**	**.006**
Diabetes	− 2.15	− .56	.574	**− .24**	**− 2.57**	**.010**
Time since diagnosis	− .27	− 1.05	.294			
RAAS medication	2.60	.55	.583	.10	.83	.410
Diuretics	**− 9.31**	**− 2.47**	**.014**	**− .23**	**− 2.44**	**.015**
NYHA class III	.73	.20	.842	**− .44**	**− 4.71**	**< .001**
LVEF	**.52**	**2.04**	**.042**	− .008	− 1.3	.194
Aspirin	*5.73*	*1.73*	*.085*	.05	.55	.582

*GFR*_*creat*_ glomerular filtration rate of creatinine, *LVEF* left ventricular ejection fraction, *NYHA* New York Heart Association, *RAAS* renin-angiotensin-aldosterone system.  Boldfaced numbers represent significant differences (*P*<.05), while italic numbers represent trends (*P*<.10).

*More than primary school

Hb concentrations were stable across the 12-month time period (*F*(1,357.31) = 1.68, *P* = .195). The overall level of self-care was not associated with Hb in both unadjusted (*F*(1,717.90) = .25; *β* = − .06, *P* = .618) and adjusted models (model 3: *F*(1,382.83) = .19; *β* = − .02, *P* = .662). Positive within-person change in self-care was associated with a trend improvement in hemoglobin in both the unadjusted (*F*(1,336.79) = 3.11; *β* = .04, *P* = .079) and fully adjusted model (*F*(1,341.54) = 3.20; *β* = .04, *P* = .075). Significant covariates of poorer Hb concentrations in the final model were NYHA function class III, presence of diabetes, prescribed diuretics, lower BMI, and female sex. There were no significant interactions between time and self-care. In the final model, the random intercept was significant (Wald = 9.35, *P* < .001), with a variance estimate of .43 (se = .05).

Time effects differed per type of cytokine. In unadjusted models, there was no significant effect of time for TNF-α (*F*(1,237.39) = 1.85, *P* = .186), while IL-6 decreased significantly (*F*(1, 212.10) = 10.34, *P* = .002) and IL-10 increased (*F*(1,238.09) = 18.41, *P* < .001), indicative of less Th2 inflammatory activity. There were no significant between- and within-subject effects of global self-care on the levels of any of the cytokines in both unadjusted (data not shown) and adjusted mixed models. In the covariate models, higher age was associated with increased TNF-α and IL-6, and presence of diabetes with higher concentrations of TNF-α, IL-6, and IL-10.

### Individual Self-Care Behaviors and Pathophysiological Outcomes

**Fig 2:**
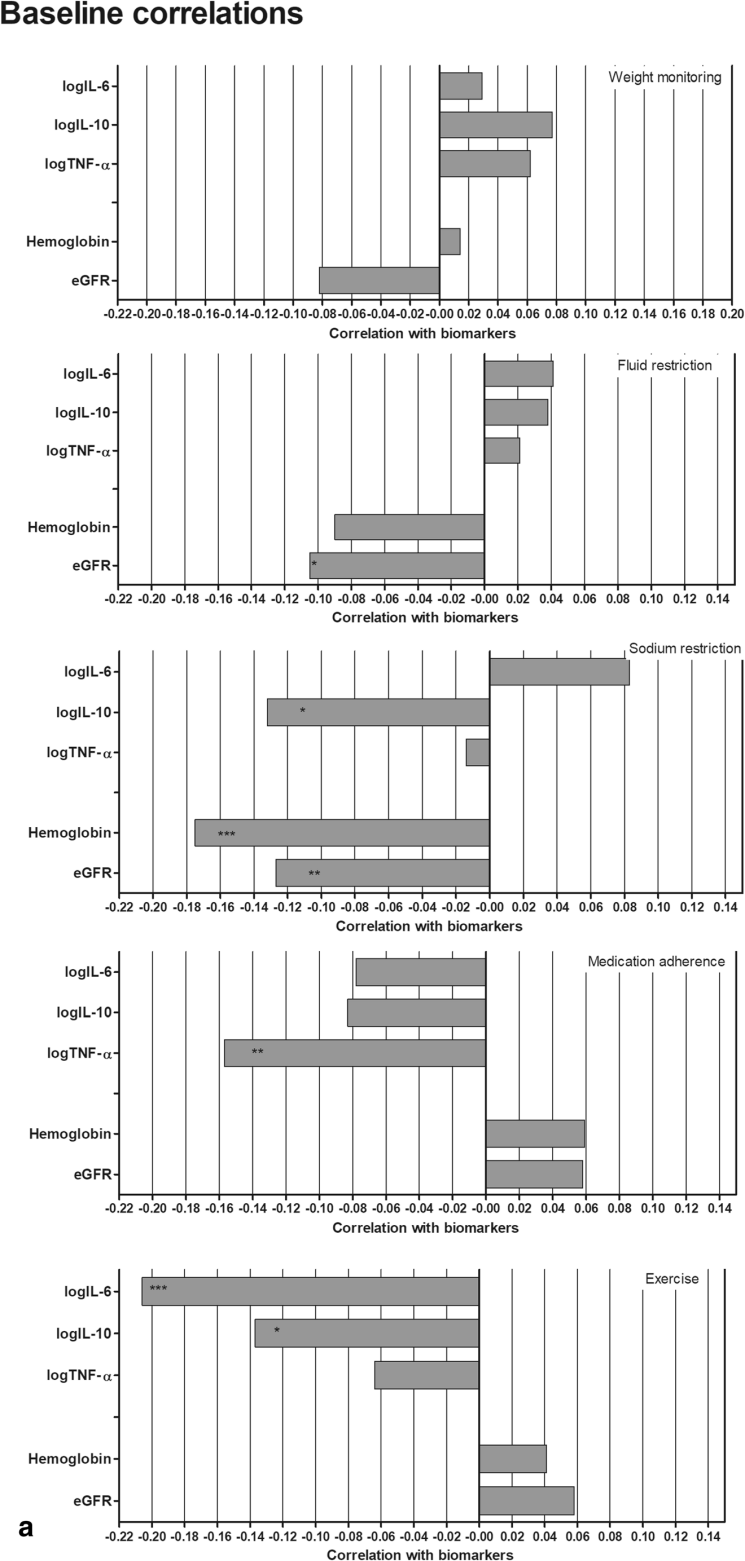

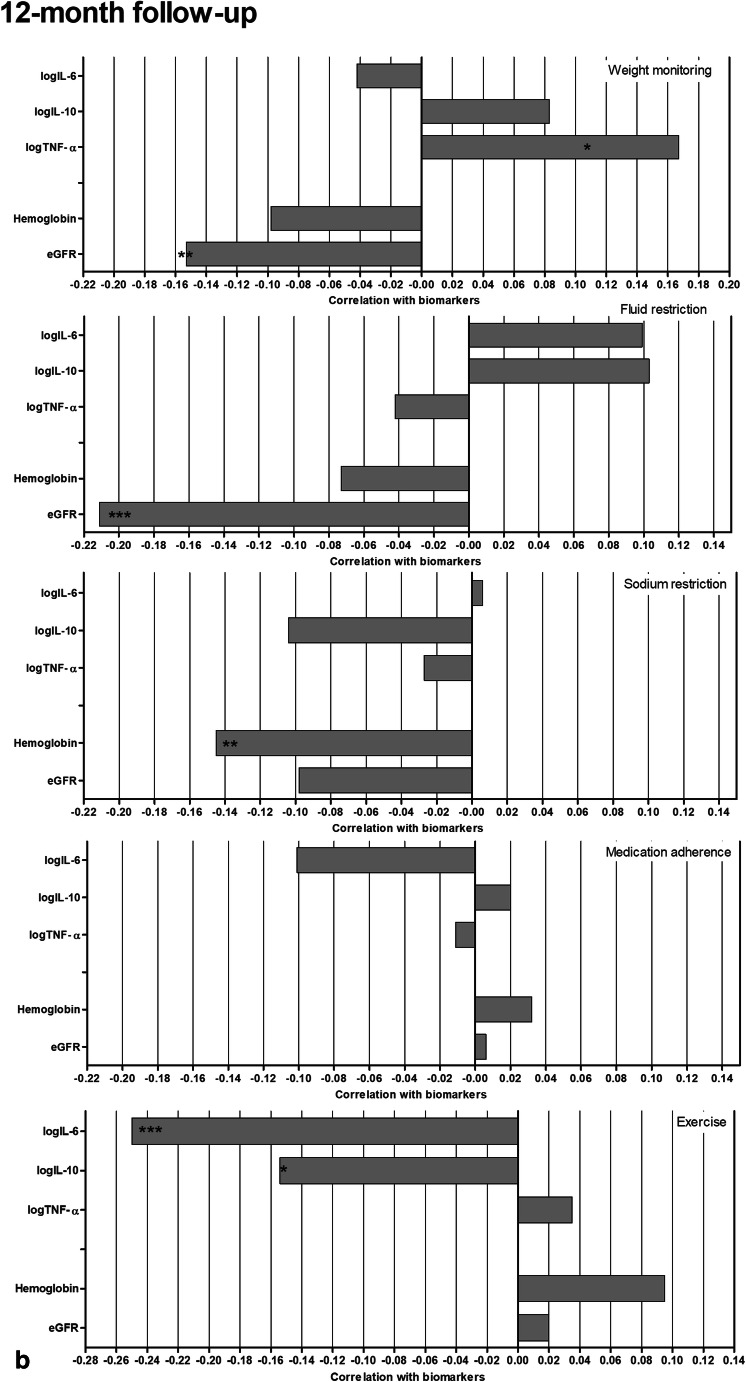
Pearson correlations between individual self-care behaviors and pathophysiological outcomes at baseline (**a**) and 12-month follow-up (**b**)

Pearson correlations of the individual self-care behaviors with the pathophysiological outcomes at baseline and 12-month follow-up are presented in Fig. 2. Adherence to the medication regime and being physically active regularly were correlated with lower cytokine levels and higher GFR_creat_ and Hb. Increased weight monitoring, fluid and salt restriction correlated with increased inflammatory activity and poorer renal and hematological function on one or both of the measurement occasions.

Next, multivariable linear mixed models were run for each pathophysiological marker to examine the association with the self-care behaviors while adjusting for the same covariates as tested in the models for the global self-care score (Table 5). Correlations among the individual self-care items were moderate (*r* = .07–.40), therefore not violating the assumption of risk for multicollinearity by testing all behaviors in one model.

**Table 5:** Multivariable associations of individual self-care behaviors and continuous levels of GFR_creat_, hemoglobin, logTNF-α, logIL-6, and logIL-10 in linear mixed models

	GFR_creat_			Hemoglobin			LogTNF-α			LogIL-6			LogIL-10		
	*B*	*t*	*P*	*B*	*t*	*P*	*B*	*t*	*P*	*B*	*t*	*P*	*B*	*t*	*P*
Daily weighing_Between_	**− 3.69**	**3.01**	**.003**	− .01	− .31	.75	.008	1.08	.280	− .003	− .26	.797	.01	.83	.405
Daily weighing_Within_	*− 1.02*	*1.69*	*.092*	.02	.71	.48	*.02*	*1.87*	*.063*	− .007	− .59	.558	.02	.99	.325
Limiting fluids_Between_	**− 3.87**	**2.75**	**.006**	− .003	− .07	.943	− .002	.23	.817	.003	.29	.772	.02	1.12	.263
Limiting fluids_Within_	**− 1.35**	**2.47**	**.014**	.01	.47	.638	< .001	.05	.964	− .008	.71	.479	− .01	1.02	.309
Low sodium intake_Between_	*− 2.5*	*1.83*	*.068*	**− .08**	**− 2.22**	**.027**	**− .02**	**2.58**	**.011**	− .008	.62	.535	**− .03**	**2.20**	**.029**
Low sodium intake_Within_	.44	.76	.446	− .04	− 1.39	.164	.004	.50	.617	.02	1.777	.079	− .002	.13	.894
Med. adherence_Between_	2.77	.83	.406	.05	.66	.510	**− .06**	**− 3.47**	**.001**	**− .08**	**− 2.74**	**.007**	− .009	.24	.813
Med. adherence_Within_	.15	.18	.861	.08	1.34	.103	− .001	.11	.911	− .008	.41	.685	− .01	.48	.631
Regular exercise_Between_	− 1.37	.94	.346	− .02	− .53	.593	− .006	.67	.506	**− .05**	**3.69**	**< .001**	− .03	1.77	.078
Regular exercise_Within_	**2.32**	**3.17**	**.002**	**.10**	**2.58**	**.010**	.001	.10	.917	− .13	.79	.431	− .01	.61	.546
Consulting_Between_	− .07	− 1.19	.233	< .001	.05	.961	< .001	1.52	.130	< .001	.65	.514	< .001	.97	.332
Consulting_Within_	**− .06**	**− 2.16**	**.032**	.003	1.63	.105	< .001	− .23	.815	< .001	.54	.593	.001	1.56	.122

Numbers represent results from the final model, including the following covariates: age, sex, NYHA function class, LVEF, partner status, education, smoking status, body mass index, diabetes, time since diagnosis, and prescribed medication. To reduce the chance of false discovery by multiple testing (subscale and individual behaviors), we applied the Benjamini-Hochberg procedure to the *P* values [24]. Boldfaced *P* values represent significance after BH correction. None of the significant *P* values became non-significant. Boldfaced numbers represent significant differences (*P*<.05), while italic numbers represent trends (*P*<.10).

*GFR*_*creat*_ glomerular filtration rate of creatinine, *IL* interleukin, *TNF-α* tumor necrosis factor alpha

Higher overall adherence to daily weighing was associated with a poorer overall kidney function (GFR_creat_: *F*(1,363.87) = 9.05; *β* = − .17, *P* = .003), while there was no significant effect of change daily weighing on change in GFR_creat_ (*F*(1, 306.81) = 2.86; *β* = − .02, *P* = .092). Limiting the daily consumption of fluids has both a significant between- and within-subject effect on GFR_creat_ (*F*_between_(1362.48) = 7.58; *β* = − .15, *P* = .006; *F*_within_(1304.20) = 6.09; *β* = − .03, *P* = .014 respectively).

Multivariable results showed that increases in daily weight monitoring over time showed a trend association with increases in TNF-α (*F*(1,209.62) = 3.50; *β* = .08, *P* = .063). Lower levels of sodium intake were significantly associated with lower overall concentrations of Hb (*F*(1,379.78 = 4.94; *β* = − .10, *P* = .027), TNF-alpha (*F*(1,257.26 = 6.64; *β* = − .14, *P* = .011), and IL-10 (*F*(1,266.54 = 4.85; *β* = − .07, *P* = .029). Change in sodium intake was unrelated to change in these biomarkers. In contrast, a generally better adherence to regular physical exercise was associated with lower levels of IL-6 (*F*(1,258.84) = 13.64; *β* = − .21, *P* < .001), and improvement in regular physical exercise was significantly associated with improvements in kidney function (i.e., higher GFR_creat_; *β* = .04, *P* = .002) and in Hb (*β* = .06, *P* < .001). Positive change in consulting behavior was associated with a reduction in creatinin clearance (*β* = − .03, *P* = .032).

The person mean of medication adherence was negatively related to overall levels of TNF-alpha (*F*(1,256.44) = 12.05; *β* = − .19, *P* = .001) and IL-6 (*F*(1,269.05) = 7.52; *β* = − .15, *P* = .007). Improvement of medication adherence over time though was not associated with changes in any of the markers in multivariable analyses. Random intercepts were all significant.

With respect to interactions with time, which were tested in the final models, results showed few effects. The between-subject effect of adherence to daily weighing was different for IL-6 at baseline vs. at follow-up (*F*(1,216.88) = 4.77, *β* = .07, *P* = .030, *B* = .03, se = .01), the effect at follow-up being larger. Also for between-subject effects of medication adherence on kidney function (*F*(1,306.34) = 7.30, *β* = .04, *P* = .007, *B* = 4.3, se = 1.6) and TNF-alpha (*F*(1,253.67) = 5.515, *β* = .08, *P* = .006, *B* = .05, se = .02), we observed a similar interaction with time. Positive changes in daily weighing significantly interacted with time to affect hemoglobin (*F*(1,348.54) = 4.47, *β* = .09, *P* = .035, *B* = .25, se = .12), and positive changes in fluid restriction significantly interacted with time to affect kidney function (*F*(1,362.15) = 4.59, *β* = .12, *P* = .035, *B* = 9.8, se = 4.6) and IL-6 (*F*(1,250.20) = 5.73, *β* = − .12, *P* = .017, *B* = − .10, se = .04). The effects of consultation behavior, salt restriction, and exercise did not interact with time.

## Discussion

To our knowledge, this is the first prospective study that examined the association of HF self-care and important pathophysiological processes related to inflammation, cardiorenal, and hematological function in patients with chronic HF. We found a prospective association between better global self-care and worse renal function beyond other clinical covariates, such as HF disease severity and diabetes. Significant within-subject effects of self-care were found for GFR_creat_ for the total scale and for behaviors important for renal function, i.e., daily weighing and limiting fluids. This means that when patients’ self-care behaviors improved over time, their renal function became poorer. Global self-care was unrelated to Hb or inflammatory markers. In line with the literature [25–29], regular physical exercise was associated with less inflammation and an improvement in exercise to improvement in hematological and renal function. In contrast, fluid and sodium restriction were associated with poorer renal and hematological function. Low sodium intake was associated with lower TNF-alpha and IL-10.

Against initial expectations, we found no evidence for a relation between global self-care (i.e., the total score) and levels of pathophysiological markers with the exception of renal dysfunction. A possible explanation may be that self-care as assessed by the EHFScB-9 does not represent a homogeneous construct, especially not in the context of explanatory mechanisms and HF outcomes. While the psychometric reliability for the total self-care scale is good, this seems most contributable to the high internal consistency of the consultation subscale. When examining the associations of the individual self-care behaviors with levels of pathophysiological markers, distinctive and even contrasting patterns were found that possibly serve as an explanation for the lack of associations found for global self-care.

Our findings are consistent with the notion that HF signs and symptoms may play an important role in driving self-care in chronic diseases [9, 30]. The necessity of performing health behaviors to monitor and manage symptoms becomes relevant or perhaps is instructed by health care providers once HF pathogenic function starts to worsen. This is in line with the findings reported by Lee et al. who found that HF patients performing well with regard to self-care management were at increased risk for myocardial stress and inflammation [9]. Patients who improved their self-care in our study did so across the board and particularly in terms of daily weight monitoring and fluid and salt restriction, which may become especially relevant when HF progresses. Improvement in fluid restriction was associated with a deterioration of renal function in our study. From these and our current findings, it should be examined whether patients need to alter their self-care habits in an earlier, perhaps preclinical phase of their disease. It is possible that increased self-care in patients with HF involves an appropriate response to disease progression and thus represents a clear marker of worsening clinical condition. Clinicians may want to train patients in good self-management before the clinical condition worsens, and before self-care is motivated by symptom severity.

Regular physical exercise was consistently associated with more favorable levels and improvement of pathopathophysiological markers beyond clinical parameters, emphasizing the importance of physical activity to maintain health in HF [29]. In contrast, weight monitoring and fluid restriction were associated with cardiorenal dysfunction, and sodium restriction with lower Hb concentrations. Counterintuitively, though supported by literature [29–31], our results may also suggest a potential harmful effect of extensive dietary restricting behaviors on levels of pathophysiological markers in patients with HF. It is unclear whether all self-care behaviors are equally beneficial within each phase of HF disease progression and/or for every patient. Given that chronic HF is a complex systemic disease, patients varying in disease severity may respond differently to self-care behaviors. Importantly, recommendations on the level of sodium restriction have been mainly based on research among patients with hypertension [30] and to a lesser extent on patients with HF. Recent studies increasingly show that the amount of sodium intake and HF disease severity are important factors to consider in terms of long-term prognosis. For example, a < 3 g/day sodium intake was only beneficial in terms of prognosis in patients with advanced HF (NYHA function class III or IV) and not in patients with mild HF [32, 33]. Another study showed that (too) strict sodium restriction of < 1.8 g/day was associated with activation of neurohormones and cytokines, and body dehydration, thus worsening outcomes in patients with advanced HF [34]. In our current study, fluid and sodium restriction and prescribed diuretics were associated with worse pathophysiological function, and positive change in behavior actually was associated with a worsening. While we assessed self-care by means of self-report, our findings conform to the results from studies that assessed fluid and sodium restriction with objective measures, e.g., with 24-h urine collection logs. A critical examination of the potential harms and benefits of fluid and sodium restriction and diuretic dose in terms of long-term prognosis may be needed while considering the different stages of HF disease severity in future research. Also, for clinical practise, these results suggest to be mindful to the patient context in which one prescribes salt restriction and to what extent.

Renal dysfunction, neurohormonal and pro-inflammatory activation, and anemia are complexly interacting systems in the pathogenesis of HF [12]. With respect to cytokines, IL-6 concentrations affect TNF-α (−) and IL-10 (+) concentrations. Pro-inflammatory cytokines, in turn, are inversely associated with Hb concentrations and contribute to anemia via multiple mechanisms. For example, TNF-α and IL-6 inhibit erythropoietin production in the kidney, as well as the function of erythroid progenitor cells in bone marrow [35–37]. These mutual interactions may also affect their relations with self-care, which may explain the lack of a unified finding. Future research is needed to examine these potential interacting mechanisms.

Reporting to perform regular physical exercise was robustly associated with less inflammation, and a positive change in exercise behavior to improvement in hematological and renal function conforms prior literature [25–29]. Cause and effect are as yet not clear. There has been a study suggesting that weight loss is mediating the association between exercise and reduced inflammation [38].

In accordance with previous findings, several socio-demographic and clinical covariates of pathophysiological outcomes were found. Diabetes, a well-known risk factor for HF disease progression [39–41], was associated with increased inflammation and poor hematological function. Increasing age was associated with increased systemic inflammation and renal dysfunction. Higher NYHA functional class, female sex, and prescribed diuretics were associated with lower Hb concentrations which has been reported previously (e.g., [12, 28]). Other significant predictors of renal dysfunction were lower LVEF and prescribed diuretics. Increasing BMI was associated with increased systemic inflammation, but also with better hematological function. This latter finding fits the “obesity paradox” in HF, a phenomenon showing that overweight and mild/moderate obesity are associated with a mortality benefit with complex underlying pathophysiological processes [42].

Current findings should be interpreted in light of several limitations. First, self-care was assessed by self-report, which is an easy and preferred clinical assessment method, but may not reflect actual behavior. Moreover, individual behaviors were reported as the degree to which patients agreed they performed the ideal self-care behavior (e.g., *I weigh myself every day, I perform regular exercise*). The limitation to these kinds of questions is that frequency of the behavior often remains unclear. Self-report measures are complicated by systematic biases and it is recommended to include multiple measurement strategies to assess self-care behavior [43]. Also, we used single items to examine individual self-care behaviors. While there are several limitations to the use of single-item measures, they are reliable as health measures [44]. As blood collection started at a later time point within the study data collection, cytokine levels were assessed in a subsample of patients who were characterized by better cardiac function. We took these clinical differences into account by including cardiac disease severity in our multivariable analyses. We, however, did not examine random slopes, i.e., testing that different patients have different biomarker trajectories over time. Future studies may want to examine this individual-specific variation in pathophysiological function over time in a sample with a longer follow-up period and more assessment occasions in a denser pattern than the current study. With respect to the random effects analysis, we only estimated random intercepts, but no random slopes, as we had no a priori hypothesis on patient-specific slopes in the association of self-care and biomarkers. This decision not to fit random slopes could have resulted in an inflated type-I error rate when random slopes would be present [45]. Future research may want to examine random slopes for groups of patients though (e.g., improving vs. stable vs. progressing patients). Finally, patients with complete data were in better cardiac condition and with fewer comorbidities than patients who dropped out, while baseline self-care scores were similar. The retention of a healthier sample in the study may have led to an underestimation of the effect of self-care on biomarker levels at follow-up.

In conclusion, our findings suggest global self-care and improvement in global self-care were associated with poorer and deteriorating renal dysfunction respectively (i.e., lower GFR_creat_) in a large cohort of patients with chronic HF, indicating that the level of self-care may reflect an appropriate response to disease progression. No associations were found between global self-care and respectively Hb concentrations and inflammation.

Performing self-care behaviors such as regular exercise and medication adherence was associated with improved physiological functioning, while restriction of fluid and sodium, and the associated daily weight monitoring were associated with adverse levels of pathophysiological biomarkers.

## Abbreviations

EHFScB: European Heart Failure Self-care Behaviour scale
GFR_creat_: Estimated glomerular filtration rate of creatinine
HF: Heart failure
Hb: Hemoglobin
IL-6: Interleukin-6
IL-10: Interleukin-10
LVEF: Left ventricular ejection fraction
NYHA: New York Heart Association
TNF-α: Tumor necrosis factor alpha
